# Assessment of durable chemoimmunotherapy response via circulating tumor DNA in advanced esophageal squamous cell carcinoma

**DOI:** 10.1111/1759-7714.14610

**Published:** 2022-08-23

**Authors:** Dongyang Yang, Fei Xu, Ying Li, Xiaorong Lai, Bohong Xian, Pengli Yu, Rongrong Chen, Zijun Li, Dong Ma

**Affiliations:** ^1^ Secondary division of Medical Oncology, Cancer Center Guangdong Provincial People's Hospital, Guangdong Academy of Medical Sciences Guangzhou China; ^2^ Department of Medicine Geneplus‐Beijing Institute Beijing China; ^3^ Department of Gastroenterology Guangdong Provincial People's Hospital, Guangdong Academy of Medical Sciences Guangzhou China

**Keywords:** chemoimmunotherapy, circulating tumor DNA, durable response, esophageal squamous cell cancer

## Abstract

Immune checkpoint inhibitor (ICI)‐based therapies have shown promising advances for the first‐line treatment of advanced or metastatic esophageal cancer (EC). However, few studies concerning the identification of patients who achieve durable response from ICIs have been previously reported. In the present study, pre‐ and on‐treatment plasma circulating tumor DNA (ctDNA) were analyzed in 10 patients with advanced esophageal squamous cell cancer (ESCC) receiving first‐line chemoimmunotherapy. Patients with decreased molecular tumor burden index (mTBI) >7% experienced longer progression‐free survival (PFS) and durable clinical benefit (DCB, PFS ≥ 6 months). In addition, five patients showed stable disease at first scan, all three patients with decreased mTBI > 7% achieved DCB, while two cases with decreased mTBI ≤ 7% experienced non‐DCB. Our results demonstrate that ctDNA monitor might help identify which ESCC patients respond to chemoimmunotherapy.

## Graphical abstract

We collected peripheral blood samples before and during first‐line chemoimmunotherapy in patients with stage IV esophageal squamous cell cancer. Molecular tumor burden index (mTBI) was calculated based on ctDNA. Patients with decreased mTBI >7% achieved longer progression‐free survival than patients with decreased mTBI ≤7%.

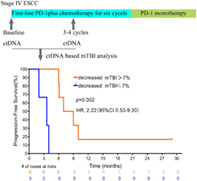

## INTRODUCTION

Immune checkpoint inhibitor (ICI)‐based therapies have recently shown remarkable promising advances for the first‐line treatment of advanced or metastatic esophageal cancer (EC).^1^, ^2^, ^3^ Several PD‐1 inhibitors, including pembrolizumab, nivolumab, camrelizumab and toripalimab have been approved for advanced EC in combination with chemotherapy regardless of PD‐L1 expression level.^1^, ^3^ However, only a minority of patients benefit from ICI‐based therapy. Therefore, the discovery of more precise biomarkers to classify patients who may achieve long‐lasting response to immunotherapy is crucial.

Liquid biopsies provide a noninvasive method to detect tumor‐derived mutations in the body fluids of cancer patients. Plasma genotyping of circulating tumor DNA (ctDNA) has shown predictive value in assessing recurrence risk in early and localized EC patients.^4^, ^5^ A recent study has shown the early dynamics of ctDNA predict first‐line chemotherapy responses for patients with esophageal squamous cell carcinoma (ESCC).^6^ However, few studies have explored the possibility of serial ctDNA analysis for prediction of durable response in ESCC patients receiving ICIs. In this study, we sought to explore whether ctDNA dynamics would enable prediction of response to first‐line ICI‐based therapy in advanced ESCC.

## METHODS

### Patients and samples

Patients with stage IV ESCC who had not received systemic treatment were drawn from Guangdong Provincial People's Hospital. Eligible patients were ≥ 18 years of age with histologically confirmed stage IV ESCC, had received PD‐1 blockade together with chemotherapy as first‐line therapy, had an Eastern Cooperative Oncology Group (ECOG) performance status score of 0 or 1, adequate organ and hematological functions, could provide baseline (before initiation of therapy) and/or had on treatment peripheral blood samples available for ctDNA testing. A total of 10 ml of peripheral blood was collected from each patient prior to treatment, and after two to three cycles of therapy. Patients all signed a written consent form prior to the study. Study protocol was approved by the institutional review boards of Guangdong Provincial People's Hospital (KY‐Q‐2022‐027‐01). According to the Response Evaluation Criteria in Solid Tumors (RECIST), version 1.1, we evaluated the clinical response of all patients two to three cycles after treatment initiation (3 weeks/cycle) or whenever there were symptoms or signs indicating disease progression.

### Identification of ctDNA with next‐generation sequencing

We performed targeted next‐generation sequencing (NGS) of 1021 genes frequently mutated in ESCC (Supplementary Table 1) and other solid tumors. Somatic mutations were identified by paired analysis of plasma ctDNA and germline DNA in blood cells. DNA extraction, library preparation, hybrid capture, sequencing, and analysis were performed as previously described.^7^ In brief, targeted capture sequencing required a minimal mean effective depth of 100× in plasma DNA. Genomic alterations, including single nucleotide variants (SNV), small insertions and deletions (indels), copy number alterations (CNA), and gene fusions/rearrangements were detected with GATK, MuTect (version 1.1.4) and BreakDancer, respectively.

### Molecular tumor burden analysis

We defined the molecular tumor burden index (mTBI) based on a comprehensive analysis of somatic alterations in ctDNA, considering tumor heterogeneity and dynamic evolution, as previously described.^7^, ^8^, ^9^ The process of mTBI calculation has been carefully described by Yi et al.^9^ The ΔmTBI was calculated based on the difference of mTBI between two ctDNA samples collected at different time points.

### Statistical analysis

Kaplan–Meier survival plots were generated for mTBI at baseline and ΔmTBI using log‐rank tests. All statistical analyses were performed with SPSS (version 21.0; STATA) or GraphPad Prism (version 9.0; GraphPad Software) software. Statistical significance was defined as a two‐sided *p*‐value of <0.05.

## RESULTS

Overall, 10 patients with metastatic esophageal squamous cell carcinoma were enrolled in this study. The clinical features and first‐line treatments for all patients are shown in Table 1. A total of six, three and one patient received pembrolizumab, camrelizumab and sintilimab based combined therapy, respectively. Treatment‐related adverse events of grade 3 or higher occurred in three patients.

**TABLE 1:** Clinical features and first‐line treatments of patients

No	Gender	Age	History of smoking	Weight (kg)	ECOG performance status	Sites of metastatic	ctDNA detection timepoints	First‐line treatment
P1	Male	75	No	62	1	Multiple lymph nodes in neck and supraclavicular, left adrenal gland metastasis	Baseline，precycle IV	Sintilimab plus docetaxel and cisplatin
P2	Male	69	Yes	59	1	Bone, posterior wall of left ches	Baseline，precycle IV, precycle VII,	Pembrolizumab plus cisplatin and 5‐fluorouracil
P3	Male	68	No	60	1	Multiple enlarged lymph nodes on both sides of the supraclavicular, left tracheoesophageal groove, retroperitoneum	Precycle IV, precycle III in monotherapy,	Pembrolizumab plus cisplatin and 5‐fluorouracil
P4	Male	50	No	58	1	Multiple lymph nodes in the right supraclavicular and right upper mediastinal space	Baseline，precycle IV, precycle VI	Pembrolizumab plus cisplatin and 5‐fluorouracil
P5	Male	54	No	64	1	Nodules in the right lower lung dorsal segment, left lower lobe basal segment	Baseline，precycle V	Camrelizumab plus docetaxel and cisplatin
P6	Male	63	No	57	1	Nodules in the mediastinum, left neck, upper and lower left lung	Baseline，precycle IV	Pembrolizumab plus cisplatin and 5‐fluorouracil
P7	Male	61	No	55	1	Mediastinum, upper lobe of both lungs, middle right lung	Baseline，precycle IV	Pembrolizumab plus cisplatin and 5‐fluorouracil
P8	Male	56	Yes	56	1	Right thoracic entrance, mediastinum, left hilar, around the cardia, hepatogastric space	Baseline，precycle III	Camrelizumab plus docetaxel and cisplatin
P9	Male	60	No	62	0	Lung	Baseline, precycle IV	Pembrolizumab plus cisplatin and 5‐fluorouracil
P10	Female	68	No	47	1	Enlarged lymph nodes in the right supraclavicular fossa, cardia, and left stomach	Baseline，precycle IV	Camrelizumab plus docetaxel and cisplatin

Plasma samples obtained from all patients before and during the treatment course underwent targeted NGS. Of these patients, the baseline plasma of P3 failed the NGS test. One patient (P2) had no somatic alteration detected at baseline, and eight patients had at least one alteration detected (range: 1–21 alterations). A total of 66 somatic alterations were detected in baseline samples, including 58 SNVs and eight CNVs. The most commonly detected mutation at baseline was *TP53*(6/8, 75%), as shown in Supplementary Table 2. P9 was tumor mutation burden‐high (TMB‐H) with a TMB value of 21.0 Muts/Mb.

**FIGURE 1:**
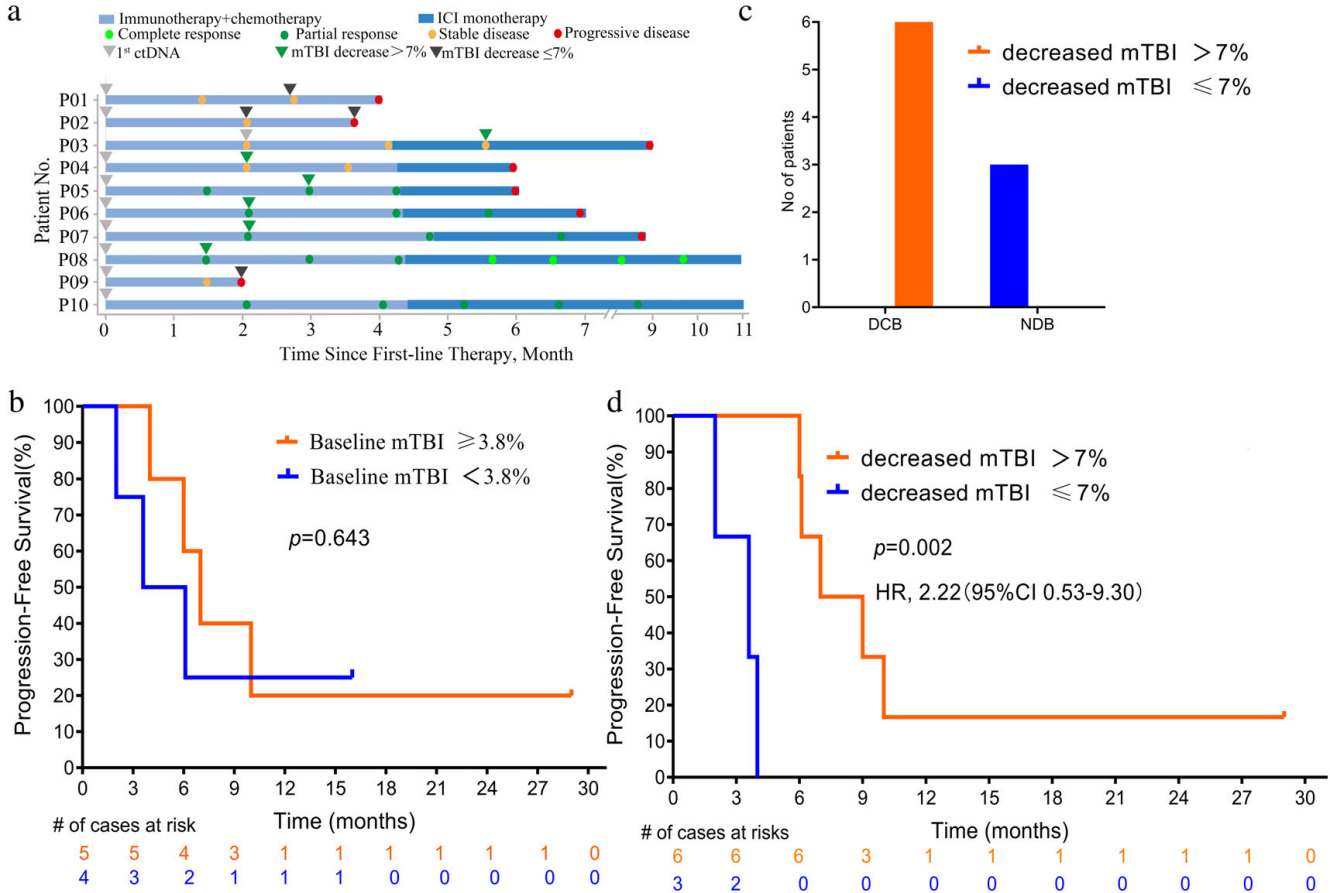
Correlation of mTBI and therapeutic response, progression‐free survival. (a) Graphic summary of ICI treatment, radiographical response and mTBI analysis. (b) Kaplan–Meier analysis of progression‐free survival in patients with mTBI equal or higher than 3.8% compared with patients with mTBI lower than 3.8% in advanced ESCC. (c) Distribution of DCB and NDB in patients with different mTBI changes. (d) Kaplan–Meier analysis of progression‐free survival in patients with decreased mTBI>7% compared with patients whose with decreased mTBI ≤ 7%. Abbreviations: DCB, durable clinical benefit; NDB, no durable benefit; ICI, immune check point inhibitor; mTBI, molecular tumor burden index; ctDNA, circulating tumor DNA

To evaluate whether changes in plasma ctDNA levels could predict a radiographical response to first‐line chemoimmunotherapy, mTBI was analyzed. A graphic summary of treatment, radiographical response and mTBI analysis are shown in Figure 1(a). Half of the patients had radiological stable disease (SD) and the remainder achieved a partial response (PR) at the first radiological scan after treatment initiation. Three experienced progressive disease (PD) during chemoimmunotherapy, while five of them experienced PD during PD‐1 monotherapy. As of August 2021, two patients remain on immunotherapy with an ongoing response.

No statistically significant difference in progression‐free survival (PFS) was observed between patients with baseline mTBI lower than 3.8 and patients with mTBI equal to or higher than 3.8 (3.8 was the median of baseline mTBI, *p* = 0.643; Figure 1(b)).

Nine patients had at least two ctDNA tests. The ΔmTBI was calculated based on the difference of mTBI between two ctDNA samples. To assess whether on‐treatment ctDNA dynamics could identify which patients will experience a durable benefit from chemoimmunotherapy in the first‐line setting, patients were classified into durable clinical benefit (DCB, complete or partial response or stable disease that lasted ≥6 months) and NDB (no durable benefit). As shown in Figure 1(c), all six patients with decreased mTBI >7% achieved DCB, while the other three patients with decreased mTBI ≤ 7% experienced NDB. Then, we explored the correlation between decreased mTBI levels and survival. The results show that patients with decreased mTBI > 7%) have longer PFS than patients with decreased mTBI ≤ 7% (median PFS was 8.0 months vs. 3.6 months, hazard ratio 2.22, 95% CI: 0.53–9.30, *p* = 0.002, Figure 1(d)).

**FIGURE 2:**
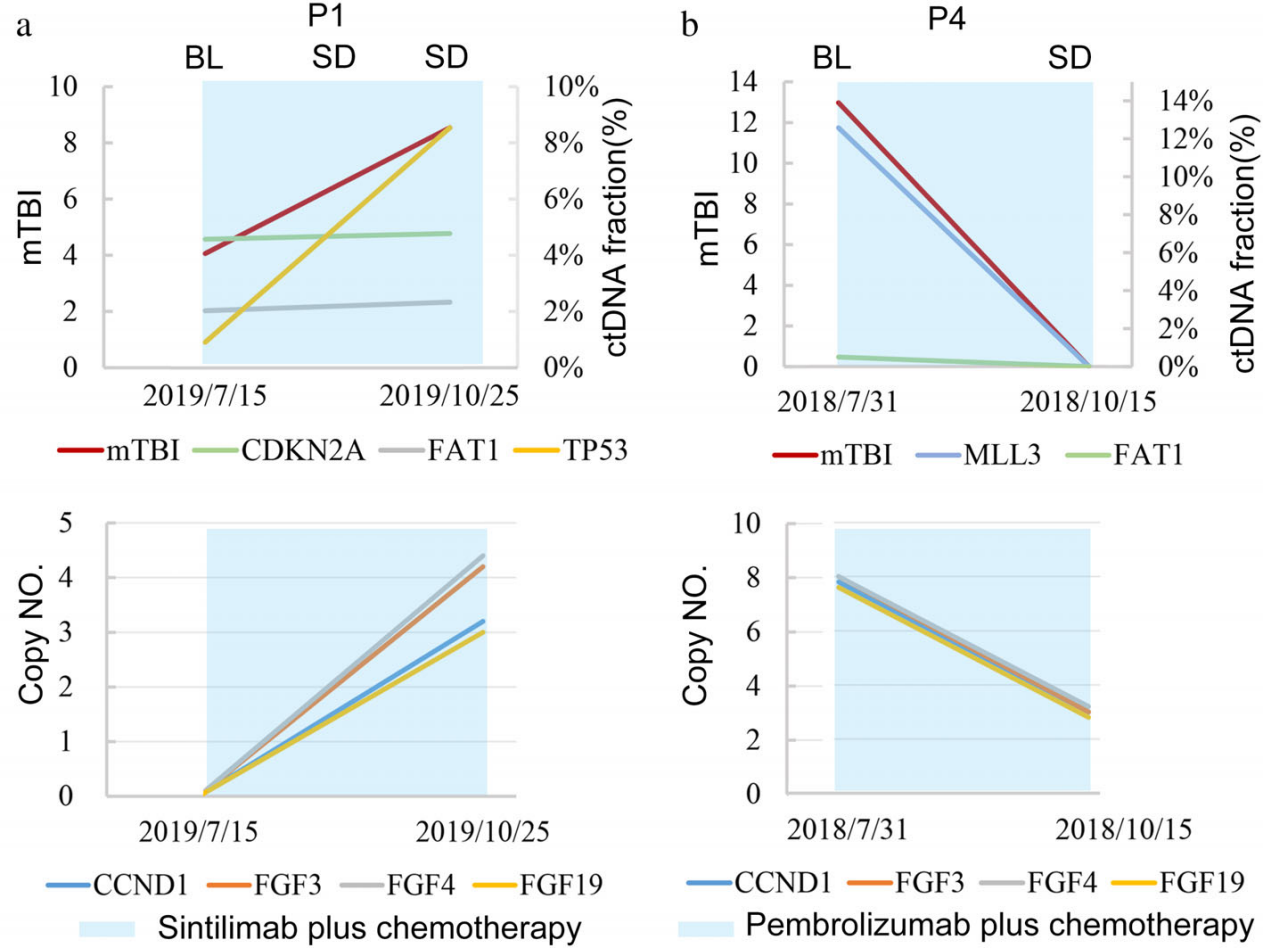
The mTBI of ctDNA before and during treatment. Top: Clinical responses and mTBI, ctDNA fractions based on baseline status. The blue line indicates mTBI results. Bottom: Changes of CNV of genes at chromosome 11q13 region detected in ctDNA. Abbreviations: BL, baseline; ctDNA, circulating tumor DNA; SD, stable disease; mTBI, molecular tumor burden index

Amplifications of chromosome 11q13 region (including *CCND1*, *FGF3/4/19*) were detected in on‐treatment sample of P1 and baseline and on‐treatment samples of P4 (Figure 2). Radiographic scan revealed SD in P1 and P4 after the start of treatment. P1 had increased mTBI, increased copy number of *CCND1* and *FGF3/4/19*, and experienced PFS of 4 months, while P4 achieved decreased mTBI > 7%, decreased copy number of CCND1 and FGF3/4/19, and had a PFS of 6 months (Figure 2). P2 and P9 had plasma ctDNA test at disease progression, no acquired mutations were found in P2, while *MST1*R and *MLL3* mutations were newly identified in P9.

## DISCUSSION

In our study, patients with decreased mTBI > 7% were more likely to achieve DCB (PFS ≥ 6 months) than patients with decreased mTBI ≤ 7%, suggesting that ctDNA monitor might help identify responders to chemoimmunotherapy. A phase 2 trial demonstrated that in HER2‐positive esophagogastric cancer, 10/13 patients with decline in ctDNA after the induction cycle of first‐line pembrolizumab and trastuzumab were progression‐free at 6 months.^10^ Similar results were found in NSCLC, which determined the role of early ctDNA changes predicting response to first‐line pembrolizumab‐based therapy.^11^, ^12^, ^13^ In those previous studies, one somatic mutation with the highest allele frequency (AF) or mean AF of all genes identified at predose was used to track ctDNA levels over time compared with baseline. mTBI was calculated based on a comprehensive analysis of ctDNA, considering heterogeneity and dynamic evolution. In breast and gastric cancer patients, mTBI had been reported to predict molecular responders to chemotherapy or target therapy.^7^, ^8^, ^9^ We first used mTBI to predict and monitor chemoimmunotherapy response. Our results demonstrate that a decrease of mTBI > 7% might help to identify those patients who respond to first‐line chemoimmunotherapy in metastatic ESCC.

In addition, the PFS of patients exhibiting stable disease varied over time as the response to ICIs can be delayed. Instead, early ctDNA dynamics may allow more accurate classification of responders compared with first radiographic imaging. In our study, five patients achieved SD as their best radiographic response, three patients with mTBI decrease >7% achieved a PFS ≥ 6 months, while two patients with mTBI decrease ≤7% experienced a PFS of <6 months, demonstrating that monitoring early ctDNA might help identify the patients who really respond to ICIs.

Several studies have demonstrated that compared with radiographic evaluation, serial ctDNA tests could predict treatment failure or recurrence earlier.^14^, ^15^ However, as a result of the lack of repeated plasma samples in the present study, we were unable to estimate the detection lead time between mTBI variations and radiographic evidence of disease progression.

Amplifications of chromosome 11q13 region have been reported to be negative predictive markers in advanced ESSC patients receiving second‐line ICI based therapy.^16^ One patient in our study had amplification of the 11q13 region and responded to ICI with PFS of 6 months, suggesting patients with this mutation may also benefit from ICI therapy. Plasma sample at disease progression of one patient with baseline TMB‐H revealed two acquired mutations, *MST1R* and *MLL3*. According to previous studies, colorectal patients with *MLL3* mutation could benefit from ICI therapy, while *MST1R* signaling promoted therapeutic resistance in *ESR1* mutant breast cancer.^17^, ^18^ Apart from mutations, the tumor immune microenvironment in ESCC, such as PD‐L1 expression on tumors and tumor‐infiltrating lymphocytes, may also potentially influence the patient's response to ICIs and should be further investigated.^19^

There are several limitations to our study. The sample size was small, there was an inconsistent timepoint of ctDNA sampling among patients, and not all patients were treated with a uniform chemoimmunotherapy regimen. Larger studies with regular ctDNA sampling are needed to determine whether mTBI decrease after chemoimmunotherapy translates into improved disease‐free and overall survival.

In conclusion, the current research indicated that a decrease >7% of ctDNA based mTBI is associated with a better prognosis and response to therapy; therefore, it might be used as a guide in patients with ESCC responding to first‐line chemoimmunotherapy. Additional studies with larger sample sizes in this specific patient population and longitudinal plasma ctDNA analysis are warranted to validate these findings.

## AUTHOR CONTRIBUTIONS

DY Yang, F Xu, D Ma, and Zj Li contributed to the study investigation,writing–review&editing. DY Yang and F Xu contributed to the writing–original draft. DY Yang, F Xu, Y Li and BH Xian contributed to the data curation. XR Lai, PL Yu and RR Chen analyzed the data. D Ma, and Zj Li contributed to the supervision.

## CONFLICT OF INTEREST

The authors declare no conflict of interest.

## Supporting information


**Appendix S1** Supplementary Tables
